# Seeing the whole picture: Inflammatory bowel disease complications and extraintestinal manifestations on cross‐sectional imaging

**DOI:** 10.1002/ueg2.12619

**Published:** 2024-07-05

**Authors:** Maria Manuela Estevinho, Nurulamin M. Noor

**Affiliations:** ^1^ Department of Gastroenterology Unidade Local de Saúde Gaia Espinho Vila Nova de Gaia Portugal; ^2^ Department of Biomedicine Unit of Pharmacology and Therapeutics Faculty of Medicine University of Porto Porto Portugal; ^3^ Department of Gastroenterology Cambridge University Hospitals NHS Foundation Trust Cambridge UK; ^4^ Department of Medicine University of Cambridge School of Clinical Medicine Cambridge UK

**Keywords:** B2, B3, Crohn's disease, CTE, EIMS, extraintestinal manifestations, fistulizing, IBD, inflammatory bowel disease, IUS, MRE, stricturing

Computed tomography (CT) and magnetic resonance (MR) enterography techniques are widely used for detection and monitoring of intestinal complications in inflammatory bowel disease (IBD). According to international recommendations, patients newly diagnosed with Crohn's disease (CD) and those with symptomatic small bowel disease should undergo small bowel assessment ideally using MR enterography, capsule endoscopy, or intestinal ultrasound (IUS).^1^ While the diagnostic yield of the three techniques is similar, cross‐sectional tools such as CT imaging (involving ionizing radiation) should be reserved for those with a suspicion of more urgent pathology such as obstructive or fistulizing disease.^2^ Although commonly used for luminal indications in IBD, the use of cross‐sectional imaging specifically for identifying extraintestinal manifestations (EIMs) has been less explored.^3^, ^4^

In a real‐world retrospective study, *Vuyyuru et al*. analyzed the prevalence of transmural complications (stricture/fistula) and the incidental finding of EIMs in patients with IBD who underwent CT or MR enterography over 9 years at two Canadian centers.^5^ The study included over 550 IBD patients, 91% of whom had CD, with a median disease duration of 11 years. In this cohort, transmural imaging identified a B2 (stricturing) or B3 (fistulizing) phenotype in more than 40% of individuals. The overall prevalence of EIMs was 25%, with one‐third in patients previously undiagnosed at the time of the enterography. Among those newly diagnosed with EIMs (*n* = 41), the most common were cholelithiasis (63%), followed by sacroiliitis (24%) and primary sclerosing cholangitis (5%).

These results corroborate current international guidance, highlighting the importance of cross‐sectional imaging to identify transmural complications. Additionally, this study emphasizes the need for vigilance and of the possibility to use cross‐sectional imaging tools to help identify EIMs.

It is important to note that for some patients the presence of EIMs may be earlier in development or asymptomatic. This may explain why there was a relatively large proportion of patients with newly diagnosed EIMs, despite a median disease duration of 11 years. In this regard, the authors demonstrate promise for cross‐sectional imaging to detect even subclinical EIMs. More timely detection and diagnosis of EIMs may be crucial for some diseases such as primary sclerosing cholangitis due to its association with malignancy and need for surveillance. Moreover, earlier detection may also enable better understanding of the burden of EIMs and help guide treatment selection.^6^ For example, earlier introduction of biologic therapy might be considered in a patient with only mild luminal IBD but who has concomitant sacroiliitis.

Although this study provides novel and important insights in a real‐world setting, there remain some unanswered questions. First, selection of the most “appropriate or optimal” cross‐sectional imaging modality remains a challenge. With increasing availability of IUS, it may be more difficult to justify use of CT or MR for first‐line cross‐sectional imaging. There are multiple potential benefits of IUS including being:less expensive, faster to perform, well tolerated for patients, with no ionizing radiation risk as well as more recently being validated to identify B2 and B3 phenotypes. However, this should be balanced with an awareness that MR in particular has been reported to have a higher specificity than IUS for small bowel disease extent, to detect deep‐seated or pelvic fistulas, as well as abscesses.^7^, ^8^ Moreover, it is unknown whether IUS could also help identify EIMs with the same level as demonstrated by CT and MR imaging in this study.

Second, the inter‐observer agreement for identifying and grading EIMs remains unclear, with a possibility for more incidental or non‐diagnostic findings if all cross‐sectional imaging were to routinely report on presence or absence of EIMs. Third, it is not clear if the additional time and acquisition costs for specific sequences dedicated for EIMs should be routinely included for all patients with IBD or just those who have specific symptoms, signs, or investigation results to warrant additional assessment. Fourth and linked to the previous point, the prognostic impact of subclinical EIMs identified on cross‐sectional imaging remains unknown. For example, while it is plausible that active joint inflammation would associate with IBD and later outcomes, the associations with other EIMs such as those of the biliary tract may not be so clear.^9^

It is important to note that prior studies have demonstrated high levels of complicated disease on cross‐sectional imaging even at diagnosis.^10^ In line with these previous findings, use of cross‐sectional imaging earlier in the disease course may provide crucial information on both IBD phenotype and the presence of EIMs. Performing such a detailed initial assessment could help better guide management decisions, potentially reducing future disability and improving quality of life for patients.

## AUTHOR CONTRIBUTIONS

Maria Manuela Estevinho wrote the initial manuscript draft. Nurulamin M. Noor provided critical input. Maria Manuela Estevinho and Nurulamin M. Noor approved the final version of the manuscript.

## CONFLICT OF INTEREST STATEMENT

NMN has received personal fees from BMS, Galapagos, Janssen, Lilly, SBK Healthcare, Takeda outside the submitted work and grants from Celltrion, Dr Falk, Pfizer, Pharmacosmos, Tillotts Pharma outside the submitted work.

## FUNDING INFORMATION

NIHR Cambridge Biomedical Research Centre, Grant/Award Number: NIHR203312

## Data Availability

Data sharing is not applicable to this article as no new data were created or analyzed.
